# Evidence of weak conscious experiences in the exclusion task

**DOI:** 10.3389/fpsyg.2014.01080

**Published:** 2014-09-23

**Authors:** Kristian Sandberg, Simon H. Del Pin, Bo M. Bibby, Morten Overgaard

**Affiliations:** ^1^Cognitive Neuroscience Research Unit – Center of Functionally Integrative Neuroscience, Hammel Neurorehabilitation and Research Centre – Danish Neuroscience Center, Aarhus University HospitalAarhus, Denmark; ^2^Institute of Cognitive Neuroscience, University College LondonLondon, UK; ^3^Department of Communication and Psychology, Center for Cognitive Neuroscience, Aalborg UniversityAalborg, Denmark; ^4^Department of Biostatistics, Aarhus UniversityAarhus, Denmark

**Keywords:** consciousness, exclusion paradigm, perceptual awareness scale, subliminal perception, unconscious processing

## Abstract

Exclusion tasks have been proposed as objective measures of unconscious perception as they do not depend upon subjective ratings. In exclusion tasks, participants have to complete a task without using a previously presented prime. Use of the prime is taken as evidence for unconscious processing in the absence of awareness, yet it may also simply indicate that participants have weak experiences but fail to realize that these affect the response or fail to counter the effect on the response. Here, we tested this claim by allowing participants to rate their experience of a masked prime on the perceptual awareness scale (PAS) after the exclusion task. Results showed that the prime was used almost as often when participants reported having seen a “weak glimpse” of the prime as when they claimed to have “no experience” of the prime, thus suggesting participants frequently have weak (possibly contentless) experiences of the stimulus when failing to exclude. This indicates that the criteria for report of awareness is lower (i.e., more liberal) than that for exclusion and that failure to exclude should not be taken as evidence of complete absence of awareness.

## INTRODUCTION

Although it is more than a 100 years since Sidis (1898) used subjective measures of visual perception, the last decade has brought about an increased scientific interest in the topic. The main question in this line of research is how conscious experience is optimally measured behaviorally. One popular method is to examine the relationship between the accuracy with which some task is performed and indications from a measure of awareness.

The measure of awareness can be relatively direct and openly subjective – e.g., asking participants to report their conscious experience (e.g., Ramsøy and Overgaard, 2004; Lau and Passingham, 2006; Overgaard et al., 2006; Rounis et al., 2010). However, conscious information is typically defined as the information that can be used for overt behaviour or higher-order cognition, and some measures utilize this connection. Some measures require participants to judge their confidence in being correct (a higher-order decision; e.g., Dienes et al., 1995), and other measures ask participants to place wagers on the correctness of their reply (a process presumed to be guided by the information available for higher-order cognition; e.g., Persaud et al., 2007). Recently, several studies have been conducted comparing these types of awareness measures as will be shown below.

When comparing different measures of awareness, researchers have used several approaches. One popular method is the “subjective threshold” or “dissociation” approach. Here, participants typically perform a forced-choice detection or discrimination task and subsequently rate their experience of the stimulus. Unconscious processing, in this case, is presumed to be responsible for any above-chance performance found when stimuli are below the so-called subjective criterion (i.e., when participants claim to have no experience of the identity of the stimulus; Snodgrass and Shevrin, 2006).

The ideal subjective measure should detect all relevant conscious knowledge (or all experiences; Merikle, 1982; Reingold and Merikle, 1988, 1990; Merikle and Joordens, 1997). Exhaustiveness, i.e., the degree to which conscious processing is detected (Reingold and Merikle, 1988), has been compared between scales in previous studies. Typically, a scale is considered more exhaustive if it indicates less unconscious processing by the guessing criterion (as explained above) and/or more conscious processing by the zero correlation criterion (a measure of how well awareness ratings predict task accuracy; Dienes et al., 1995; Sandberg et al., 2010). Unfortunately, the problems associated with poor exhaustiveness cannot be solved simply by preferring the scale that shows the greatest sensitivity as some scales misclassify unconscious information as conscious – that is, they are not exclusive (Reingold and Merikle, 1988). Generally, the solution is to compare scales for which there is no *a priori* reason to assume a difference in exclusiveness.

Using this approach, one study found that post-decision wagering (PDW) was generally inferior to confidence ratings (CR) in an artificial grammar paradigm because PDW was affected by loss aversion (Dienes and Seth, 2009). Another study replicated this finding for visual identification, but also found that ratings on the perceptual awareness scale (PAS) were more exhaustive than CR (Sandberg et al., 2010). This finding was replicated in a very recent study (using a gender identification task), but only when awareness ratings were made after the identification task (the study found that most scales indicated less awareness when used before the identification task; Wierzchoń et al., 2014). PAS has also been found to be more exhaustive than dichotomous ratings of awareness (Overgaard et al., 2006). Based on these studies and more, we have previously ventured the hypothesis that the most exhaustive measure may simply be asking participants directly about their experience (Overgaard et al., 2010; Overgaard and Sandberg, 2012), and that especially PDW is best used when participants are unable to use a more direct measure (e.g., in studies with non-human animals; Sandberg et al., 2013). Generally, we tentatively propose that whenever participants report their awareness indirectly, whatever task they perform, it introduces an extra process that might fail, or it requires participants to be able to link the quality of experience with task performance flawlessly.

Nevertheless, examining the relationship between task accuracy and different types of awareness ratings is not the only method for examining unconscious processing, and it may indeed be claimed that the core problem of any subjective rating approach is to rule out weak conscious perception explanations, i.e., that what appears to be subliminal, in fact, is an effect of weak conscious perception (c.f. Snodgrass, 2002). One way to avoid subjective ratings altogether is by using so-called exclusion tasks (Jacoby et al., 1993; Debner and Jacoby, 1994; Jacoby, 1998). Here, participants are asked to solve an experimental task without using information from a briefly presented prime. Participants thus compare their candidate response with the prime (Jacoby, 1998; Visser and Merikle, 1999), and the reasoning is that overt exclusion of the prime is possible as long as it is consciously perceived, but if the prime (or some aspects of it) were perceived unconsciously, it influences performance and produces above-chance relative match frequencies. Although it has recently been argued (Persaud and McLeod, 2014) that the method fulfills all criteria for an optimal measure of awareness as proposed by Newell and Shanks (2014), it is presently an open question how participants rate their awareness when failing to exclude and whether such exclusion approaches less vulnerable to weak conscious perception explanations than PAS. It is imperative for the validity of exclusion tasks that no conscious perception is found when exclusion fails following the assumptions set up by Merikle and Joordens when explaining the meaning of exclusion failure:

“The fact that the immediately preceding words were used as responses despite the instructions not to use the words suggests that masked words were perceived without awareness, at least on some proportion of the trials. This interpretation follows from two critical assumptions. First, conscious perception of the immediately preceding word leads subjects not to use it to complete the stem. […] Second, responses are controlled by conscious influences whenever either conscious influences alone or both conscious and unconscious influences are present”

(Merikle and Joordens, 1997, p. 113).

It is thus stated that conscious perception takes priority so that whenever conscious perception is present, participants exclude successfully, and exclusion failure is thus an indicator of the (complete) absence of awareness. However, if consciousness is graded or continuous, some potential problems occur, namely the problem of the presence of weak conscious perception. In this context, it has been claimed that exclusion tasks share the potential problems of subjective threshold approaches (Snodgrass, 2002; Snodgrass and Shevrin, 2006). The criticism has been formulated from a single-process signal detection theory perspective, but one does not need to accept this view in order to consider or accept the criticism. The main argument behind the criticism is that sensory evidence in general is continuous, and the participant will only exclude the candidate word on a given trial if the evidence exceeds a certain threshold. This necessitates that a criterion is set for when to exclude, and from the single-process signal detection theory perspective there is no guarantee that this criterion reflects anything but a decision with its own criterion. From a less radical perspective, it may simply be argued that the criterion for exclusion is not necessarily the most exhaustive, and it may indeed be possible that participants weak experiences before they exclude, just as they report weak experiences on the PAS before they report them on a dichotomous scale or before they report any confidence in being correct (Overgaard et al., 2006; Sandberg et al., 2010). This is an empirical question, and examining whether participants have weak experiences when they fail to exclude (thus rejecting the second premise of Merikle and Joordens) is possible. This is related to the exhaustiveness of exclusion tasks.

In this context, it should be emphasized that there are several aspects to exhaustiveness. One aspect that we have highlighted above is whether exclusion failure is vulnerable to the weak conscious perception criticism, i.e., whether participants report weak experiences when they fail to exclude, and this aspect is critical for the validity of the use of exclusion tasks in isolation. Another aspect is the general level of metacognition indicated by each measure – i.e., whether awareness ratings or exclusion performance indicate the most unconscious processing. This last question is difficult to answer without using the process dissociation procedure (PDP) to estimate unconscious processing based on both inclusion and exclusion task performance, and this aspect is thus beyond the goal of the present study. The goal here is to examine whether exclusion failure should be accepted as evidence for the complete absence of awareness.

In the present study, we specifically examined the relationship between exclusion failure and awareness ratings by asking participants to perform an exclusion task and subsequently rate their awareness of the prime using the PAS. We hypothesized that if exclusion requires less sensory evidence than reporting weak experiences, above-chance failure to exclude should be observed only for the lowest awareness rating, and awareness ratings are thus likely to be affected to the weak conscious perception criticism (i.e., participants are unwilling/unable to report weak experience that they nevertheless use to guide overt behavior). If, however, above-chance failure to exclude is also observed when participants report some awareness of the prime, participants require less sensory evidence to report weak experiences than to exclude (and the weak perception criticism thus applies to the exclusion task). This would mean that exclusion failure should not be taken as evidence for the complete absence of conscious perception and that the second assumption put forward by Merikle and Joordens (1997) is not true. The result would also generally support the claim that failure to exclude is sometimes a matter of not trusting weak perception enough to use it to exclude or that the weak perception is so poor that it simply cannot be used (we will return to this issue in the Discussion).

## MATERIALS AND METHODS

### PARTICIPANTS

Sixteen healthy participants (nine females) with normal or corrected-to-normal vision gave informed consent to participate in the experiment. The mean age was 24.9 years (SD = 1.67). The local ethics committee, De Videnskabsetiske Komitéer for Region Midtjylland, provided written confirmation that no ethical approval was required for the study according to Danish law, specifically Komitéloven §7 and §8.1.

### APPARATUS AND STIMULI

Stimuli were generated and displayed in Spyder 2.1.11^1^ using Python 2.6.6^2^ and PsychoPy v.1.74.01^3^. They were displayed on a 14^′′^ LED monitor with a screen resolution of 1366 × 768 at a refresh rate of 60 Hz. Stimuli consisted of 308 four to six letter Danish words, and masks were rows of six pseudorandom letters. Words were found using a Danish frequency dictionary, and it was confirmed that at least two four to six letter words could be constructed using the first three letters of any individual target word.

### PROCEDURE

**FIGURE 1:**
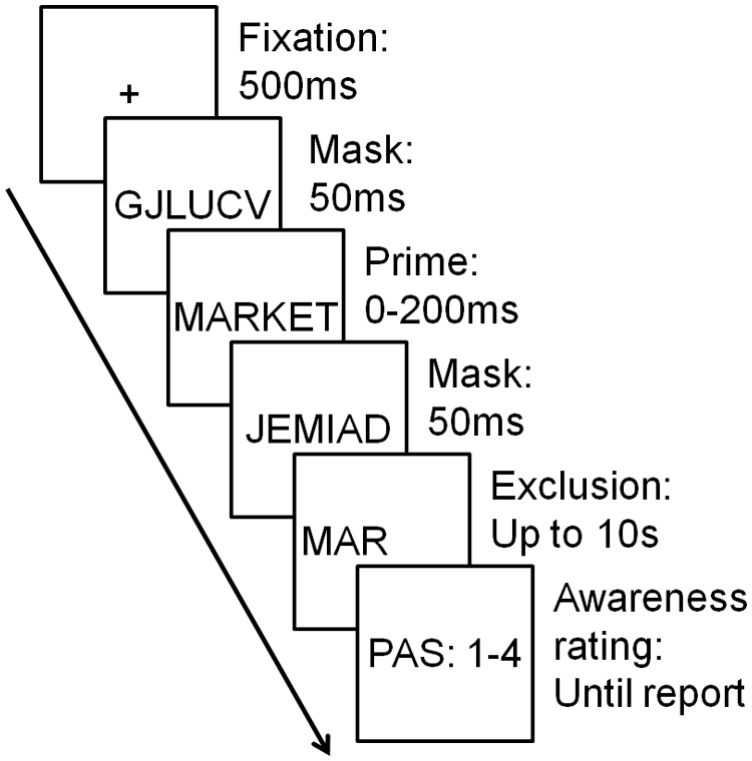
**Experimental paradigm.** A forward and backward masked prime lasting between 0 and 200 ms was presented on each trial. Following prime presentation, a three letter word stem appeared on screen and participants had to complete the word stem with any word except the prime. In the example shown here, participants should avoid writing “MARKET,” but could write “MARCH” or “MARS” for instance. After this, participants reported their awareness of the prime using the perceptual awareness scale (PAS).

Participants performed an exclusion task in which they were asked to complete a three letter word stem *without* using a briefly displayed primed word (**Figure 1**). The three letter word stem was always identical to the first three letters of the primed word. At the onset of each trial, a fixation mark appeared for 500 ms. The fixation mark was followed by a forward mask consisting of six pseudorandom letters displayed for 50 ms. Next followed the prime word with a duration pseudorandomly selected between 11 possibilities between 0 and 200 ms with no possibilities in the interval between 150 and 200 ms: {0, 16.7,..., 133.3, 150, 200}. For prime durations of 0 ms, no prime was presented. The prime was followed by a backward mask consisting of six pseudorandom letters displayed for 50 ms. A word stem consisting of the first three letters of the target word followed the backward mask and remained on screen until participants had completed the word stem or until 10 s had passed. Finally, a graphical representation of the PAS (**Table 1**) appeared on screen, and participants were asked to indicate their experience of the prime word using the keyboard number keys. The response options were: “1: No experience” (NE), “2: Weak glimpse” (WG), “3: An almost clear experience” (ACE), and “4: A clear experience” (CE). It is important to note that PAS is not simply a “labeled four-point scale” (Sandberg et al., 2013). In some publications using PAS, scale points are discussed as PAS1–PAS4 (e.g., Overgaard et al., 2006; Timmermans et al., 2010), whereas in others, they are discussed as the category labels NE, WG, ACE, CE (e.g., in Overgaard et al., 2008, 2013). Whereas this is just a matter of wording, the latter indicates more directly that PAS crucially depends on the definition of the four categories. Thus, participants were instructed that NE should be used when there is no experience at all, not even a faint sensation. WG should be used when there is a very weak/vague visual experience without any ability to specify what was perceived. ACE should be is used when there is an experience of what was perceived, yet unclear or blurry. CE should be used when there is a clear experience of what is perceived.

**Table 1:** The perceptual awareness scale (PAS).

Label	Description (from Ramsøy and Overgaard, 2004)
(1) No experience	No impression of the stimulus. All answers are seen as mere guesses
(2) A weak experience	A feeling that something has been shown. Not characterised by any content, and this cannot be specified any further
(3) An almost clear experience	Ambiguous experience of the stimulus. Some stimulus aspects are experienced more vividly than others. A feeling of almost being certain about one’s answer
(4) A clear experience	Non-ambiguous experience of the stimulus. No doubt in one’s answer

*Scale steps and descriptions.*

The experiment consisted of a practice block and six experimental blocks. For all blocks (including practice), each of the 11 prime durations was used four times in a pseudorandom order, resulting in a total of 44 trials per block. The experiment consisted of 308 trials in total for each participant.

### STATISTICAL ANALYSIS

The relationship between awareness ratings and matches of primed and reported words in the exclusion task was analyzed using logistic regression. Match (1 if the prime word was reported within the allotted time-period of 10 s and 0 otherwise) was considered as the dependent variable and awareness rating and stimulus duration along with the interaction between the two as independent variables. A random participant effect was also included in the analysis. Likelihood Ratio (LR) tests were used to assess systematic differences. Data were analysed using Stata version 12.1.

## RESULTS

**FIGURE 2:**
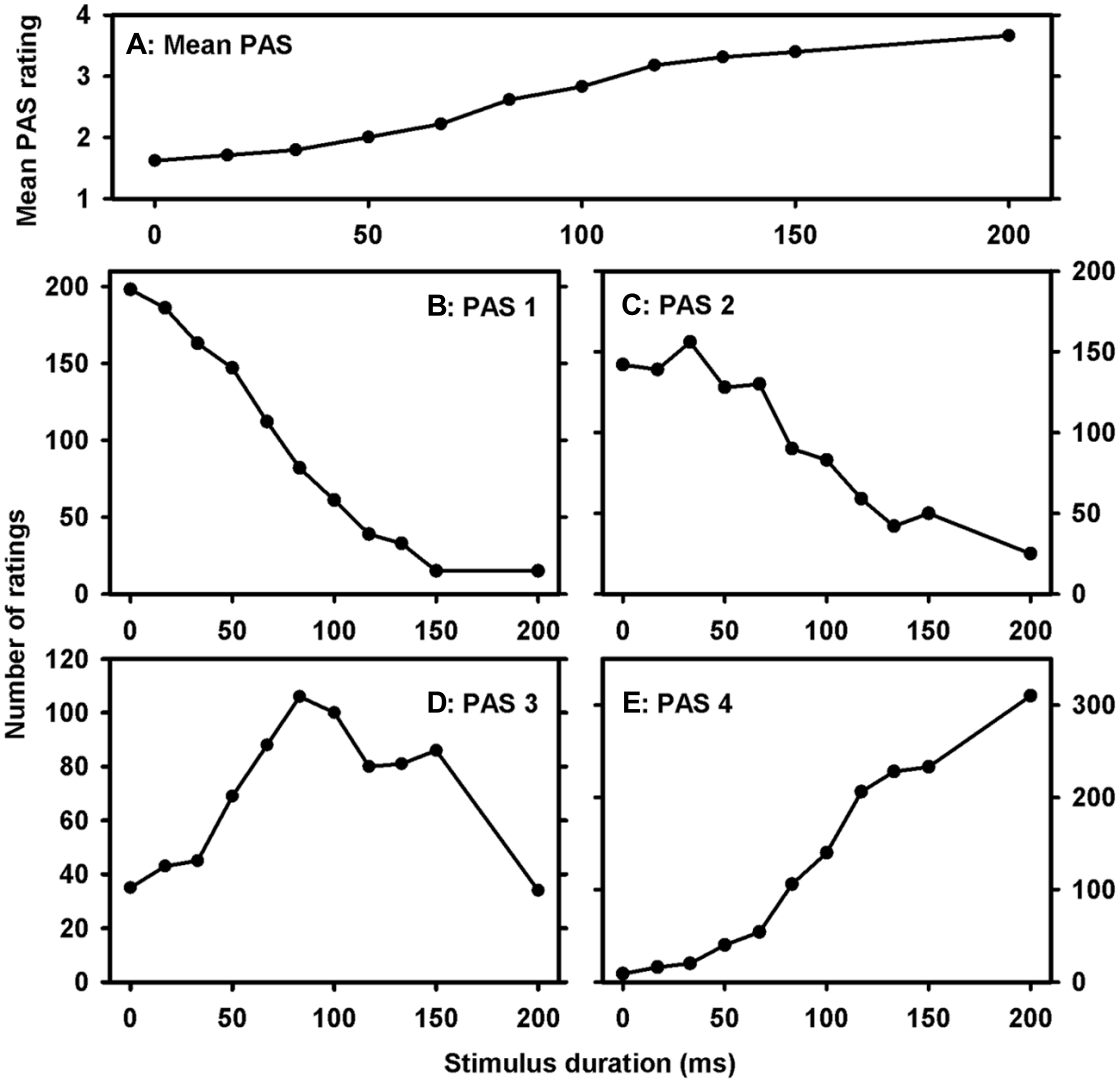
**Response distribution. (A)** Mean PAS as a function of stimulus duration. **(B–E)** Response distribution as a function of stimulus duration and PAS rating Note that each PAS rating was used across a wide range of stimulus durations and that the mode of each rating was increasing with increasing reported clarity of experience (PAS rating).

First, the relationship between objective clarity (stimulus duration) and subjective clarity (PAS rating) was examined in order to confirm that different PAS ratings reflected different conscious experiences. As seen in **Figure 2**, mean PAS rating as a function of stimulus duration appeared to have a sigmoidal shape as observed in previous PAS experiments (Sandberg et al., 2011). Furthermore, all PAS ratings were used across a wide range of stimulus durations, but for PAS1 (NE), the mode was 0 ms, for PAS2 (WG) it was 33 ms, for PAS3 (ACE) it was 83 ms, and for PAS4 (CE) it was 200 ms. In other words, when the stimulus became physically clearer, higher PAS ratings were used more frequently, thus indicating that participants used higher PAS ratings to report clearer experiences as instructed.

**FIGURE 3:**
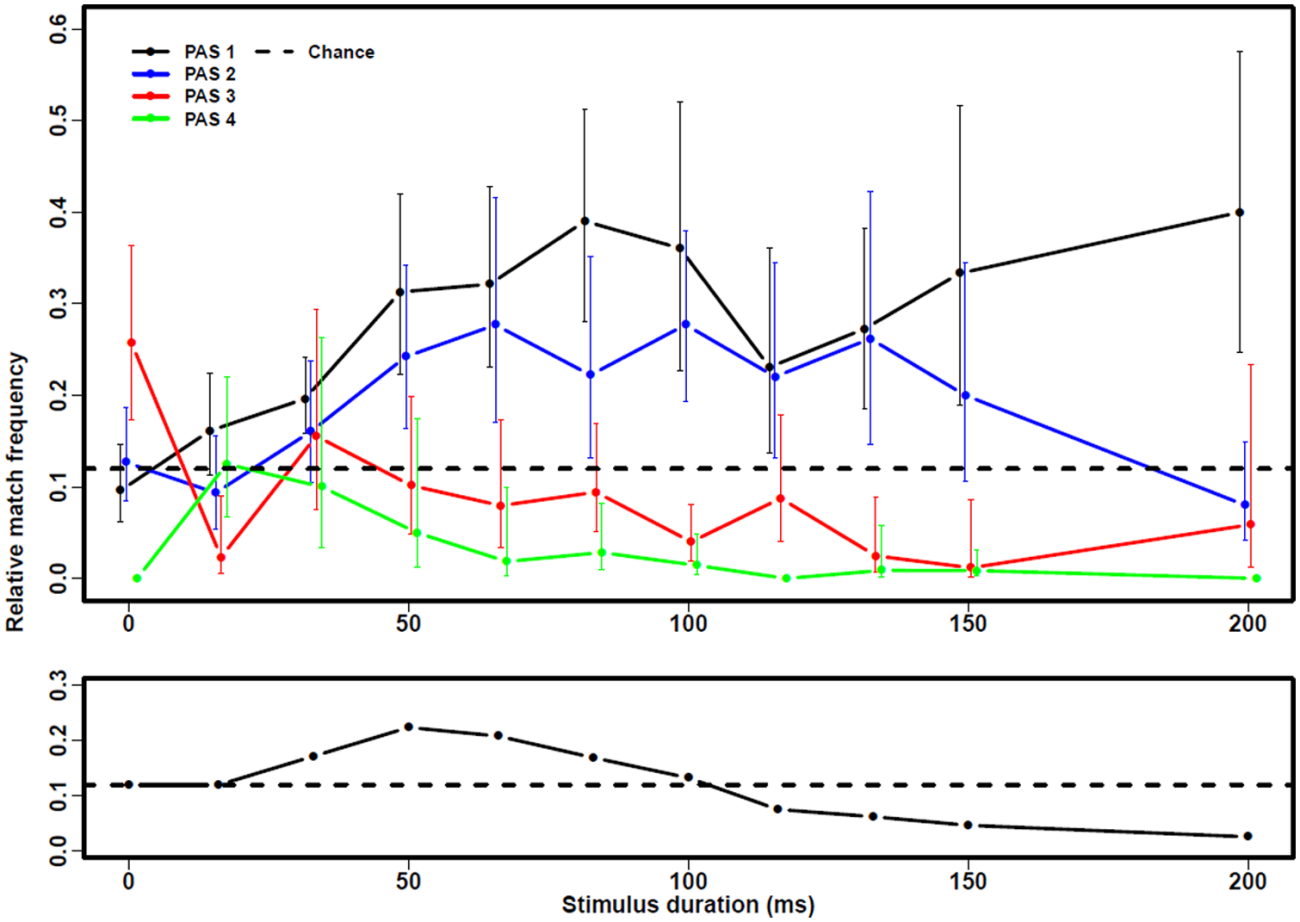
**Exclusion performance. (Top)** Match frequency (the frequency with which the prime was used to complete the word stem) with 95%-CI from the logistic regression model is plotted as a function of stimulus duration for each PAS rating PAS1 (no experience), PAS2 (a vague experience), PAS3 (an almost clear experience), and PAS4 (a clear experience). At “0 ms”, no prime was presented and match frequency here thus reflects the probability of coincidentally selecting the same word as the computer. Note that after around 33 ms, match frequency increases for PAS1-2 and decreases for PAS3-4, indicating that participants fail to exclude both when they report not having seen the prime at all, but also when they had a vague experience of it. **(Bottom)** Overall match frequency as a function of stimulus duration without taking into account PAS rating.

Next, relative match frequency was plotted for each awareness rating and overall without taking awareness rating into account as a function of stimulus duration (**Figure 3**). In general, above-chance relative match frequencies were observed for PAS1 (NE) and PAS2 (WG; χ^2^(10) > 98 and *p* < 0.0001 in both cases) whereas below-chance relative match frequencies were observed for PAS3 (ACE) and PAS4 (CE; χ^2^(10) > 230 and *p* < 0.0001 in both cases), with chance defined as the group-level relative match frequency for *t* = 0 ms (12.0%). Significant above-chance relative match frequencies at the *p* < 0.05 level were observed for PAS1 (NE) for all stimulus durations except 17 ms (*p* < 0.001 for all of these except 117 ms). For PAS2 (WG), significant above-chance match were observed for all stimulus durations in the interval 50–133 ms (*p* < 0.005 for 50, 67, and 100 ms). For PAS3 (ACE), significant below-chance relative match frequencies were observed at 17, 100, and 133–150 ms (*p* < 0.005 for 100 ms). For PAS4 (CE), significant below-chance relative match frequencies were observed in the 67–200 ms interval (*p* < 0.005 for all of these, except 67 and 133 ms).

Finally, relative match frequency was compared between PAS ratings. A small difference was observed in relative match frequency between PAS1 (NE) and PAS2 (WG; LR = 21.0∼χ^2^(11), *p* = 0.033, uncorrected for multiple comparisons). The difference was thus numerically small and not significant if corrections for six comparisons (between all PAS ratings) were made. However, relative match frequencies for both PAS1 (NE) and PAS2 (WG) were statistically significantly higher than for PAS3 (ACE) and PAS4 (CE), and relative match frequencies for PAS4 (CE) were significantly lower than for PAS3 (ACE; LR > 141.4∼χ^2^(11) and *p* < 0.0001 for all comparisons).

## DISCUSSION

Overall, the results indicated that the criterion for reporting weak experiences was more liberal than the criterion for exclusion, and it is thus unlikely that exclusion tasks are less vulnerable to weak experience critiques than ratings on the PAS. Crucially, this means that exclusion failure should not be taken as evidence of the complete absence of awareness, thus indicating that the second assumption for the validity of exclusion tasks (Merikle and Joordens, 1997) is not true. Specifically, we found that failure to exclude a primed word from report was observed almost to the same extent when participants reported that they had “no experience” of the prime (PAS1) as when they had “a vague experience” or perceived “a weak glimpse” (PAS2), but not when they had “an almost clear experience” (PAS3) or “a clear experience” (PAS4). In other words, participants failed to exclude the prime not only when they claimed to see nothing at all, but also when they had a weak experience of the target. One interpretation is that participants use only highly reliable information to guide overt behavior, yet they are nevertheless consistently able to distinguish weak experiences from the absence of experience.

It appears highly unlikely that the results were caused by PAS ratings being used randomly (i.e., that reports of awareness were unreliable) as it was confirmed that participants used PAS similarly to how it has been used in previous studies, i.e., when the stimulus was physically clearer, higher PAS ratings were used more frequently, thus indicating that participants used higher PAS ratings to report clearer experiences as instructed. These results are consistent with previous observations that PAS ratings do not only increase as a function of physical clarity, but also that each PAS rating is associated with a different accuracy level in stimulus identification (i.e., inclusion) tasks, again indicating that PAS ratings reflect different experiences (Ramsøy and Overgaard, 2004; Sandberg et al., 2010).

For these reasons, it appears more likely that the results were caused by participants being unwilling or unable to let weak (and potentially unreliable) experiences guide exclusion, or that they saw no reason to exclude the first word that came to mind because they were unaware that weak experiences influenced word generation. There are thus at least three interpretations of the results: (1) Participants could have had information that they chose not to use because they did not want to risk indicating confidence in something they were very uncertain about. (2) Participants were unable to use the information that they had. (3) Participants saw no reason to use the information they had as they were unaware that it was reliable.

The first interpretation could be hypothesized as part of the explanation for PAS has fared better than CR in some experiments as participants might fear looking over-confident if they report any confidence in cases where they only have a hunch, but the explanation is not very likely as CR are generally not affected by risk aversion (Dienes and Seth, 2009), and it thus seems unlikely that exclusion tasks should suffer from this. Additionally, in an exclusion task the conservative, risk-averse action might be to exclude even when there is only low confidence in order to avoid reporting something incorrect. This leaves the explanations of inability to use information or simply not using it because it seems irrelevant.

The definition of PAS2 ratings as reflecting subjective detection (but not identification) of a stimulus is relevant to both these interpretations. Specifically, if participants use PAS2 as instructed, they will use it to report that something was presented, but that they do not know what was presented (Ramsøy and Overgaard, 2004). Based on the current experiment, it is nevertheless not possible to distinguish the two interpretations, and we simply conclude that exclusion is typically not performed for weak, possibly contentless experiences. This means that exclusion tasks are unlikely to be a solution to any alternative weak conscious perception accounts that could be used to criticize findings of unconscious perception using the subjective threshold approach with PAS as the criterion for using PAS2 is already very liberal, and more so than for exclusion. Nevertheless, we emphasize that the results of the present experiment should be interpreted with caution as all aspects of exhaustiveness have not been examined. Specifically, it has not been examined if analyses based on PAS ratings indicate less unconscious processing across stimulus durations than the PDP. This comparison is non-trivial and would necessitate that inclusion task performance is also obtained.

Some alternative interpretations should also be discussed. One interpretation of the use of PAS2 ratings – slightly different from the “detection” interpretation – has been proposed by Dienes and Seth (2010). They argue that a given stimulus feature is either perceived or not, and that PAS2 ratings reflect ratings of perceived, but irrelevant, stimulus features. When these irrelevant features are seen, the probability of seeing a relevant feature unconsciously may nevertheless be higher than when nothing is perceived at all, and instead of PAS being more exhaustive than, for instance, CR, it is in fact less exclusive and misclassifies some unconscious information as conscious. This interpretation would also hypothetically account for the findings of the present experiment if PAS2 ratings simply reflect reports about irrelevant information, and that decisions to exclude are based only on all-or-none information about relevant stimulus features. However, the explanation has difficulty accounting for some other observations.

First, if conscious experience were indeed dichotomous, it is difficult to explain why participants consistently claim to perceive images at different levels of clarity, and why different levels of awareness are associated with different levels of task accuracy in identification tasks. A graded/partial awareness perspective (Kouider et al., 2010) can account for this to some extent, but we do not find it convincing in all cases. Graded perception of complex objects is easy to imagine, but it is more difficult when very simple objects, such as line segments only differing in orientation, are used. Here, any graded perception of even a single pixel should be diagnostic and result in peak accuracy, yet this is not observed – accuracy increases slowly as a function of awareness (Overgaard et al., 2006).

Second, if CR (or exclusion decisions) exclusively reflects perception of relevant features whereas PAS reflects perception of both relevant and irrelevant features, it should be expected that whenever ratings of full confidence are given, accuracy should be around peak level and at least as high as for PAS4 ratings (as the participants indicate complete awareness of the relevant features). However, a previous study found that at low stimulus intensities accuracy for PAS4 ratings was very high (almost at 100%) whereas it was relatively poor for CR4 ratings (around 75%, with chance at 25%; Sandberg et al., 2010). The better accuracy-awareness correlation for PAS than for CR is thus not present only for low ratings where weak experiences could lead to subliminal perception. This finding can be explained by CR4 having a lower criterion than PAS4 with all evidence being treated equally, or alternatively that participants are generally worse at using CR [e.g., these ratings reflect a different kind of knowledge (Timmermans et al., 2010)], but PAS ratings reflecting irrelevant information does not explain why participants are not accurate when reporting peak confidence.

These two issues argue against the interpretation that PAS2 ratings simply reflect irrelevant information, and in the context of the present experiment, we thus believe that the evidence weighs in favor of our original accounts. However, even if the alternative account should be true, it does not make PAS use less relevant as participants can generally distinguish these cases of weak (or subliminal) perception from cases of no experience, thus leaving it for the scientist to decide how to treat them in the analyses.

One explanation for a lower criterion for awareness ratings than for exclusion is related to a very recent study (Wierzchoń et al., 2014), which demonstrated that if visual identification is performed before an awareness rating is given, then a higher level of metacognition (i.e., a better relationship between awareness rating and accuracy) is found than when the awareness rating is given before the visual identification task. This may be taken as evidence that performing the visual identification increases metacognition, although it cannot be ruled out entirely that the effect would not be found when simply increasing the time between stimulus presentation and awareness report or when inserting any (i.e., even an unrelated) task. In any case, it could mean that part of the willingness/ability to report weak experiences while failing the exclusion task in the present experiment was caused by the effect of increased metacognition at the time of the awareness report. We do not interpret this as a confound in our study, but rather a potential explanation, as both awareness ratings and the exclusion task were used as they are typically used in the literature; i.e., exclusion tasks are never preceded by awareness ratings, but awareness ratings are almost always preceded by some kind of first order detection or identification task.

In summary, we conclude that participants consistently and reliably report weak experiences when they fail to exclude, thus demonstrating that the second assumption necessary for the validity of exclusion task is not true (the assumption being that whenever there is any conscious perception, even if there is also unconscious perception, participants will exclude). This means that the criterion for reporting weak experiences is more liberal than the criterion for exclusion, and in addition, it is thus unlikely that exclusion tasks are less vulnerable to weak conscious perception explanations than awareness ratings. For these reasons, we argue that there is no evidence in favour of using other methods for acquiring information about participants’ experience than allowing them to report it directly – whenever the state of the participants and the experimental context allow for it – and that exclusion failure should not be taken as evidence of absence of awareness. It is more likely to reflect the absence of a certain strength of conscious perception necessary for that perception to be used to guide overt behavior, not a complete absence of awareness. Nevertheless, we emphasize that the present study has not conducted a direct comparison of all aspects of exhaustiveness for awareness ratings and the PDP as a whole. The conclusions thus concern weak experience accounts and the interpretation of exclusion failure, and we encourage direct comparisons of awareness ratings and PDP in future studies.

## Conflict of Interest Statement

The Review Editor Dr Bert Timmermans declares that, despite having collaborated with authors Dr Kristian Sandberg and Dr Morten Overgaard in the past, the review process was handled objectively and no conflict of interest exists. The authors declare that the research was conducted in the absence of any commercial or financial relationships that could be construed as a potential conflict of interest.

## Footnotes

1 http://code.google.com/p/spyderlib

2 http://www.python.org

3 http://www.psychopy.org
